# PGD: a pangolin genome hub for the research community

**DOI:** 10.1093/database/baw063

**Published:** 2016-09-11

**Authors:** Tze King Tan, Ka Yun Tan, Ranjeev Hari, Aini Mohamed Yusoff, Guat Jah Wong, Cheuk Chuen Siow, Naresh V.R. Mutha, Mike Rayko, Aleksey Komissarov, Pavel Dobrynin, Ksenia Krasheninnikova, Gaik Tamazian, Ian C. Paterson, Wesley C. Warren, Warren E. Johnson, Stephen J. O'Brien, Siew Woh Choo

**Affiliations:** 1 Genome Informatics Research Laboratory, Centre for Research in Biotechnology for Agriculture (CEBAR), High Impact Research Building, University of Malaya, 50603 Kuala Lumpur, Malaysia; 2 Department of Oral and Craniofacial Sciences, Faculty of Dentistry, University of Malaya, 50603 Kuala Lumpur, Malaysia; 3 Institute of Biology Sciences, Faculty of Science, University of Malaya, 50603 Kuala Lumpur Malaysia; 4 Theodosius Dobzhansky Center for Genome Bioinformatics, Saint Petersburg State University, St. Petersburg 199004, Russia; 5 Oral Cancer Research and Coordinating Centre, Faculty of Dentistry, University of Malaya, 50603 Kuala Lumpur, Malaysia; 6 McDonnell Genome Institute, Washington University, St Louis, MO 63108, USA; 7 Smithsonian Conservation Biology Institute, Front Royal, Virginia 22630, USA; 8 Oceanographic Center, Nova Southeastern University, Ft Lauderdale, FL, 33004, USA; 9 Genome Solutions Sdn Bhd, Suite 8, Innovation Incubator UM, Level 5, Research Management & Innovation Complex, University of Malaya, 50603 Kuala Lumpur, Malaysia

## Abstract

Pangolins (order Pholidota) are the only mammals covered by scales. We have recently sequenced and analyzed the genomes of two critically endangered Asian pangolin species, namely the Malayan pangolin (*Manis javanica*) and the Chinese pangolin (*Manis pentadactyla*). These complete genome sequences will serve as reference sequences for future research to address issues of species conservation and to advance knowledge in mammalian biology and evolution. To further facilitate the global research effort in pangolin biology, we developed the Pangolin Genome Database (PGD), as a future hub for hosting pangolin genomic and transcriptomic data and annotations, and with useful analysis tools for the research community. Currently, the PGD provides the reference pangolin genome and transcriptome data, gene sequences and functional information, expressed transcripts, pseudogenes, genomic variations, organ-specific expression data and other useful annotations. We anticipate that the PGD will be an invaluable platform for researchers who are interested in pangolin and mammalian research. We will continue updating this hub by including more data, annotation and analysis tools particularly from our research consortium.

**Database URL**: http://pangolin-genome.um.edu.my

## Introduction

Pangolins are ancient creatures whose ancestors are thought to be members of a suborder of Palaeanodonta from some 60 million years ago (1). Pangolins are rare, toothless nocturnal burrowing mammals that are covered with tough, protective keratin scales over its whole body (2–4). These hard scales are used as a protective layer; when threatened, pangolins quickly roll up into a tight ball and the scales act as a shield. Pangolins are listed as endangered as reported in the IUCN red list (5) of species, because of deforestation and hunting for their meat is considered a delicacy and the scales, blood and skin are used in traditional Chinese medicine (6–8).

We have recently sequenced the genomes of two pangolin species, the Malayan pangolin (*Manis javanica*) and the Chinese pangolin (*Manis pentadactyla*) from Malaysia and Taiwan, respectively, using high-throughput next-generation sequencing (NGS) approaches. Using the NGS technology, we have also sequenced the transcriptomes of different organs of the Malayan pangolin. To further facilitate research into pangolin biology, we have implemented a Pangolin Genome Database (PGD) as a central hub for hosting genomic resources and information of pangolins, together with useful analysis tools for the research community. PGD provides interactive and user-friendly web interfaces for users to search, browse, retrieve, visualize and analyze pangolin genomic and transcriptomic data and information. The PGD will serve as a computational platform for researchers to advance research in this field, particularly in the area of conservation of this unique endangered mammalian species and also provide a useful resource for research in mammalian evolution and biology, in general.

## Database Sources

PGD currently hosts all data, results and information about pangolins generated by the International Pangolin Research Consortium (IPaRC) (website: http://pangolin.um.edu.my). Currently, we have the reference genome sequences of Malayan pangolin and Chinese pangolin. The Malayan pangolin genome was sequenced using Illumina HiSeq 2000 at BGI, Hong Kong and assembled using SGA-0.10.10 (9) (coverage = 146×). The assembled contigs were scaffolded using SOAPdenovo2 scaffolder and achieved N50 of 204 525 bp, assembled scaffold genome size of 2.5 Gbp (41). For the Malayan pangolin, we have also sequenced the transcriptomes from different organs including cerebrum, cerebellum, liver, heart, lung and thymus. The sequenced Malayan pangolin specimen was provided by the Department of Wildlife and National Parks Malaysia and sequenced and assembled by the Genome Informatics Research Group (GIRG), University of Malaya, Malaysia. The Chinese pangolin specimen was originated from Taiwan and sequenced and assembled by the Warren Research Group from the Genome Institute of Washington University, USA, and analyzed by Theodosius Dobzhansky Center for Genome Bioinformatics, St. Petersburg, Russia. The Chinese pangolin genome (coverage = 56×) sequenced using Illumina platform, assembled using SOAPdenovo v1.0.5 and scaffolded using L_RNA_scaffolder approach achieving N50 of 157 892, assembled scaffold genome size of 2.2 Gbp (41).

### Genome data and functional annotation

We annotated the entire genome of both pangolin species using a well-established MAKER pipeline, which was designed for the annotation of emerging model genomes using evidence from *ab initio* gene prediction, sequence homology approach and the transcriptomic data from Malayan pangolin (10). MAKER server is a well-established annotation pipeline for eukaryotic genomes annotation (10). First, repetitive sequences in the pangolin genomes were identified and masked using RepeatMasker (11) and RepeatModeller (12). After the repeat masking process, the RNA-Seq data from Malayan pangolin were mapped to the genomes as first evidence and the canine cDNA sequences from Ensembl (13) used as a reference for protein homology. This automated pipeline integrates the evidence from its gene prediction algorithm and produces high-quality gene models in subsequent runs. The gene models were *de novo* predicted by Augustus, GENSCAN and SNAP supported by MAKER annotation pipeline (10) using default parameters and canine cDNA sequences as reference, integrates with the homologs and RNA-Seq evidence to generate the final set of gene models. Using this stringent procedure, we identified 23 446 genes in the Malayan pangolin genome and 20 298 genes in the Chinese pangolin genome. These genes were further annotated using BLAST2GO, allowing better understanding of the function of each gene. BLAST2GO uses BLAST algorithm on the annotated protein sequence query to find homologs (14). Then, a straight forward mapping is performed to retrieve the gene ontology (GO) term with the obtained hits. In order to annotate the functional domains/signatures, different biological databases were used such as GO modulate, Go Slim (15), Enzyme Code annotation with KEGG (16) maps and also InterPro (17) annotation with the default parameters. BLAST2GO assigned functions to 21 451 (91%) genes of Malayan pangolin and 19 287 (95%) genes of Chinese pangolin (Table 1).

**Table 1.** Summary statistics of two pangolin genome and transcriptome datasets in PGD

Genome	Malayan Pangolin	Chinese Pangolin
Number of scaffolds	81,732	87,621
Estimated coverage (X)	146	56
Estimated Genome size	2,549,959,554 bp	2,205,289,822 bp
N50 (bp)	204,525	157,892
# of protein-coding genes	23,446	20,298
# of annotated genes	21,451 (91%)	19,287 (95%)
# of pseudogenes	4660	2416
# of transcripts	89,751	NA

Assembly statistic of pangolins genome. Adapted from Pangolin genomes and the evolution of mammalian scales and immunity. by Choo et al., 2016.

### Transcriptome data

PGD stores the sequences of expressed genes from different organs, namely the cerebellum, cerebrum, liver, heart, kidney, lung, thymus and spleen of the Malayan pangolin. Briefly, these transcriptomes were sequenced using an Illumina HiSeq 100 bp Paired End sequencing strategy. To generate a representative catalog of pangolin genes, the sequencing reads from all organs were pooled and *de novo* assembled using three different approaches: SOAPdenovo (18), Velvet (19) and Trinity (20) software. To generate a high-quality set of pangolin genes, we only accepted common assembled transcripts or genes predicted by the three different assemblers. The assembled data were filtered and clustered based on their similarity, and the longest transcripts were selected as unigene. This resulted in a set of 89 751 unigenes in the Malayan pangolin transcriptome (Table 1).

### Pseudogene annotation data

To the best of our knowledge, the Malayan and Chinese pangolin genomes are the first whole-genome sequencing efforts for the Pholidota order; therefore, information regarding pseudogenes are still not available. Here, PGD offers information about the putative pangolin pseudogenes. To predict pseudogenes in the pangolin genomes, we searched the whole-genome sequences using MAKER-generated protein-coding genes (parent genes) as query sequences through the whole-genome using Pseudopipe pipeline (21). Pseudopipe is a well-established pipeline for whole-genome pseudogene screening (22–24). Protein sequences derived from MAKER annotation were used as queries to BLASTALL (25) through the whole pangolin genomes. After all homologous BLAST hits were reported, Pseudopipe removed all hits that belongs to its parent genes, and merged small gaps between two short hits and output as potential pseudogene candidates. These candidate pseudogenes were then screened using the tFasty (26) tool to report insertion, deletion, premature stop codon and frame shift mutation events that may disrupt the normal functions of the genes in order to generate the final pseudogene data set. Stringent thresholds were used to filter all the false-positive candidates (1E−10 e-value, 70% parent gene coverage, 40% gene identity). All pseudogenes were reported as duplicated or processed pseudogenes based on the nature of the reported hits. There are 4660 pseudogenes reported in Malayan pangolin and 2416 pseudogenes (Table 1) in the Chinese pangolin genome after applying the stringent filtering criteria.

### PGD implementation

**Figure 1.**
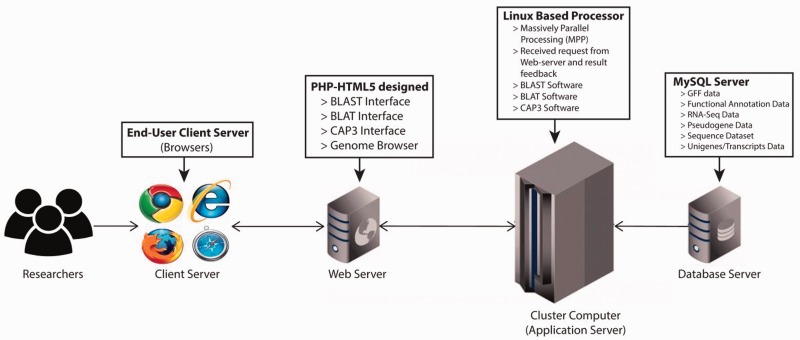
PGD four-tier web application architecture. (client workstation, web server, application server and database server).

The PGD hub was developed based on the four-tier web application architecture (client workstation, web server, application server and database server) implemented under the Linux system using various common software packages including Apache, MySQL, PHP and Perl. The website was designed in PHP-HTML5 using Codelgniter and Twitter Bootstrap as the back-end and front-end frameworks, respectively (Figure 1). In addition, the website has been separated into logic, presentation and application data into three interconnected parts following the Model-view-controller framework. For the client-side, this was achieved with jQuery, which is a feature-rich JavaScript library that may enhance user interaction with the web pages through AJAX (Asynchronous JavaScript and XML) that is used to transfer data between the client side and the server side. The genomic annotation data that are generated from in-house scripts and published software are stored in the MySQL database management system.

PGD contains four main features/tabs: (i) ‘Browse’ tab for users to browse all pangolin genome annotation and transcripts information; (ii) ‘Tools’ tab that contains a list of analysis tools for users to analyze pangolin genomic data; (iii) ‘Genome Browser’ tab that incorporates dual genome browser, UCSC genome browser and JBrowse for visualizing pangolin genome sequences and annotation and (iv) ‘Download' tab that allows users to download all pangolin genome/transcript data and annotations. In addition, PGD also has implemented quick search function, allowing users to rapidly search for genes of interest stored in the MySQL database. Furthermore, users can perform sequence searches against the pangolin genome sequences using their sequences of interest and the incorporated web-based BLAT and BLAST tools. Our web architecture allows users to access and submit their tasks using the front-end provided securely with the web server via the Internet. We normalized and optimized the database schema in order to reduce the data redundancy of pangolin's genomic data. Therefore, the database was well designed in such a way that MySQL would be performing optimally, without the loss of data integrity. As such, user can submit tasks, search, browse and retrieve the genomic data through PGD in a robust and efficient manner.

## Graphical User Interface Design

### Overview

**Figure 2.**
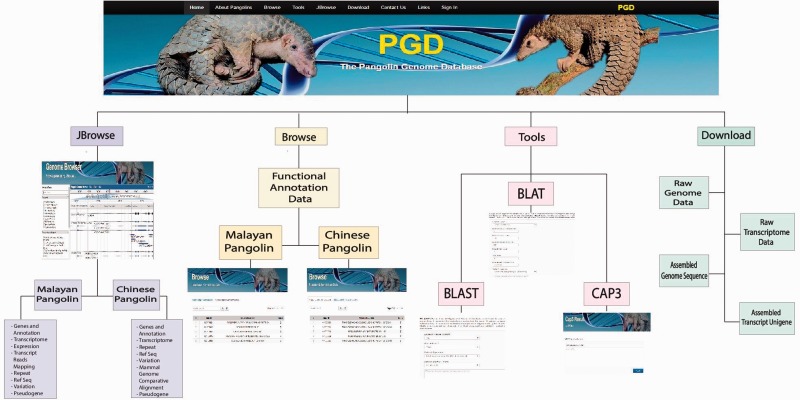
Schematic structure of the PGD.

The homepage of PGD contains general descriptions of pangolins in the main panel and manually compiled information about pangolins such as latest news and conferences, blogs and published articles in the right-side panel. On the top of the PGD homepage, several option tabs are provided, allowing users to access different features of PGD. For instance, the Browse tab allows users to quickly browse the detailed annotation data of Malayan and Chinese pangolins, as well as transcriptomic data for the Malayan pangolin. In addition, the Tools tab allows access to analysis tools such as BLAST (25) and BLAT (27) packages that we have incorporated into PGD. Users also can visualize the genome and genomic features using the unique real-time keyword search feature for fast and smooth searching of genes of interest (Figure 2).

### Browsing pangolin genomic data in PGD

**Figure 3.**
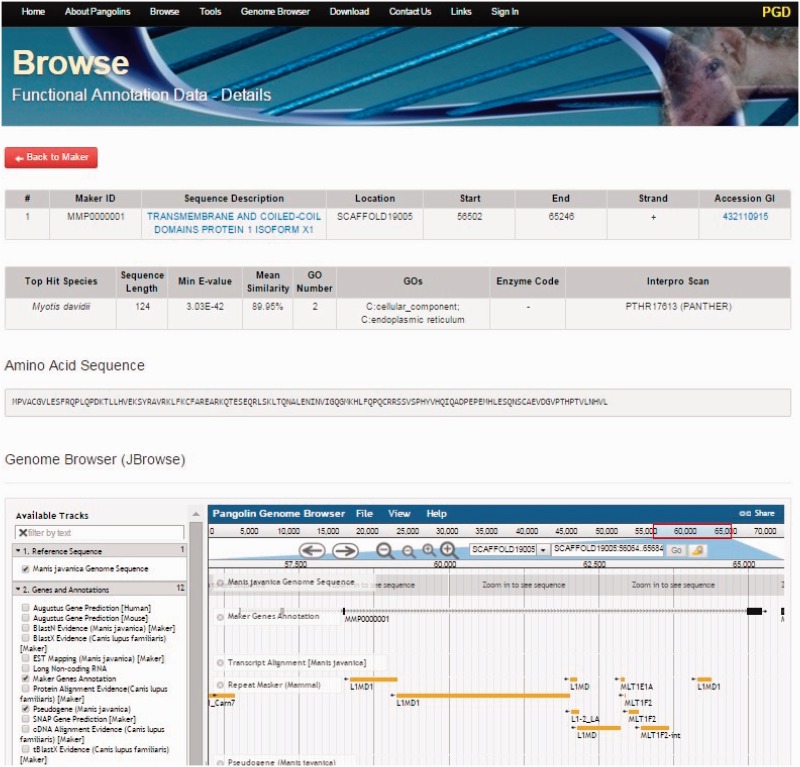
A screenshot of gene details page. This page will display information of a gene including its sequences and functional annotation.

By clicking on the Browse tab on the top of the PGD homepage, users can access all gene information of the both pangolin species (Figure 3). All genes will be displayed after users click on the tab. Users can access the gene details page containing the gene annotation and functional information of a particular gene by clicking on the ‘Details’ button associated with the gene of interest. This gene details page will display all information about the gene of interest including sequence name, scaffold ID, start and stop position of the genes, putative functions, gene sequences and BLAST2GO alignment results such as ‘Top Hit Species’, ‘Min E-value’, annotated ‘GOs’ information, ‘InterPro Scan’ domain information. JBrowse is also incorporated in this page, allowing users to visualize and explore the locus and neighboring regions of the gene within the pangolin genome.

### Keyword and sequence searches

PGD contains a vast amount of pangolin genomic, transcriptomic data and annotations. Therefore, it is necessary to provide intuitive web-based graphical user interface (GUI) allowing users to rapidly search a large volume of data. We have implemented a keyword search system in PGD to allow users to search genes of interest in a spontaneous manner. Alternatively, users can search by sequences. PGD has built-in BLAST (25) and BLAT (27) tools, which allows users to search or compare a query sequence against the pangolin genome and transcriptomic data. Using the BLAST tools, users can perform nucleotide searches to each annotated sequence (BLASTN), whole-genome nucleotide searches (BLAST Whole Genome), protein searches of each annotated sequence (BLASTP), and nucleotide searches of each annotated protein sequence (BLASTX). Users can choose the pangolin species of interest (either Malayan pangolin or Chinese pangolin) and also set the desired cutoffs (e.g. expect value and enable to search for low compositional complexity regions) for their sequence searches. Alternatively, users can perform a sequence search using BLAT, which is structured differently from BLAST, to search the similarity in a query sequence but it accepts an exact or nearly exact match to find the hit. Unlike BLAST which is a local alignment software, BLAT is also designed to map transcripts/ESTs that have no introns back to the reference pangolin genomes. In this case, users would be able to examine the location of this transcript and also its gene structure within the genome. Using our provided web-based BLAST, users are able to set the parameters such as (i) minimum number of matches, (ii) minimum score, (iii) minimum identity percentage, (iv) maximum gap, (v) tile size and (vi) maximum intron size. The BLAT outputs will be linked with the genome browser, allowing instant visual comparison of each match hit.

## Interactive Pangolin Genome Data Visualization

A fully dynamic real-time genome browser, JBrowse, is incorporated into PGD, allowing users to instantly browse and visualize pangolin genome sequence and annotation data on the fly. JBrowse provides a few useful features: (i) supports fast and smooth genome navigation, (ii) utilizes multiple types of data format, (iii) provides real-time genome browsing with light server resource required, (iv) provides high-speed visualizing the data, sequences and genome annotation results.

**Figure 4.**
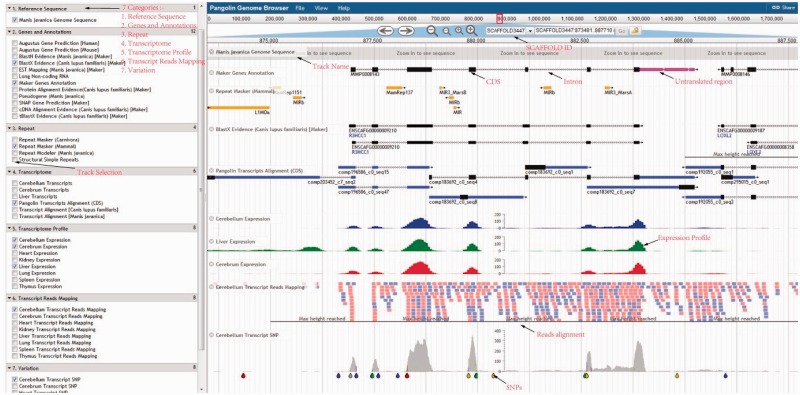
Pangolin genome browser. Users can turn on and off the annotation tracks in the left panel.

Using the pangolin genome browser (JBrowse) (Figure 4), users can choose whether to visualize the pangolin data in Malayan pangolin genome or Chinese pangolin genome. Currently more annotations (e.g. transcriptomic data) are available for the Malayan pangolin than the Chinese pangolin, because many research projects are ongoing under the IPaRC for the Malayan pangolin species. Once users choose which genome to explore, they can see all relevant annotation tracks in the genome browser. For instance, we have a basic track as the reference genome sequence of the selected genome. We also have different annotation tracks that can be triggered on/off for easier visualization and analysis. These tracks are classified into different categories such as ‘Genes and Annotations’, ‘Repeat’, ‘Transcriptome’, ‘Transcript Reads Mapping’, ‘Organ Specific Expression’ and ‘Variation’ which represent our annotations from different analyses.

### Genes and annotations tracks

This category will show the results of MAKER gene models including the evidence-based and *de novo* gene predictions, together with the predicted pseudogenes. In the pseudogene track, users can visualize the predicted pseudogenes in the selected pangolin genome. These pseudogenes are labeled with ‘PSSD’ and ‘DUP’ as categorized by PseudoPipe pipeline (21). The pseudogene tracks report all useful features found in the pseudogenes, including parent gene coverage ‘Frac’, identity ‘Ident’, insertion ‘Ins’, deletion ‘Del’, frameshift ‘Shift’ and stop codon ‘Stop’. This information will provide a better understanding of pseudogene attributes found in both pangolins.

### Repeat tracks

The repetitive element information is provided by different software which we display in three different tracks: RepeatMasker, Repeat Modeler and Structural Simple Repeat tracks. RepeatMasker track shows the repetitive elements screened in the pangolin genomes using two different repeat libraries: the carnivore repeat library and mammal repeat library. We used SSRIT (28) for scanning the simple sequence repeat or structural sequence repeat present in both pangolin genome. The SSRIT reports the entire simple sequence repeats and allows user to study microsatellites in pangolin genomes.

### Transcriptome tracks

Users can explore and visualize expressed genes in Malayan pangolin through the tracks under this category. The expressed transcripts generated by pooling all reads from the eight pangolin organs and assembled using three different assemblers are available here. These common transcripts were mapped onto the reference genome using GMAP (29). Moreover, users can also explore and examine which transcripts/genes are expressed in a specific pangolin organ such as cerebrum, cerebellum and liver through the provided organ-specific transcripts tracks.

### Transcript reads mapping tracks

Users can also examine the expression level of each gene of Malayan pangolin across different organs. Briefly, for each organ or organ-specific transcriptome, we mapped RNA-Seq reads to the Malayan pangolin genome sequence and the reads mapping data of eight different organs (cerebrum, cerebellum, liver, heart, kidney, thymus, spleen and lung) were available in the ‘Transcript Reads Mapping’ category.

### Transcriptome profile tracks

Raw transcript reads mapping results of eight different pangolin organs were normalized using RPKM value and converted into a graphical bigwig format track that visualized the expression profile of each organ. Users are able to examine the expression profiles of a gene of interest across eight different organs simultaneously. Of note, the PGD genome browser also links some features (e.g. genes and repeats) to external resources. Among of these resources are RepeatMasker database for each repeats elements, BLASTX evidence tracks is linked to well-established databases to check each hit on ENSEMBL database, PANTHER database (30), Dog Genome SNP database (31) and UniProt (32) by simply right clicking on the features in the tracks. These external database interlinks may help users understand further information of the features.

### Multiple mammalian genome structural alignment

We performed the multispecies structural alignment of two pangolin genomes to other mammalian genomes (dog, cat, cow, horse, human and mouse) using the Progressive Cactus software (33). The animal genome data used in the alignment are shown in Table 2.

**Table 2.** Genome assembly version for each mammal genome used for multiple sequence alignment

Animal	Scientific name	Genome assembly
Dog	*Canis familiaris*	CanFam3.1
Cat	*Felis catus*	Felis_catus_8.0
Cow	*Bos taurus*	Bos_taurus_3.1
Horse	*Equus caballus*	EquCab_2.0
Human	*Homo sapiens*	GRCh37.p5
Mouse	*Mus musculus*	GRCm38.p4

**Figure 5.**
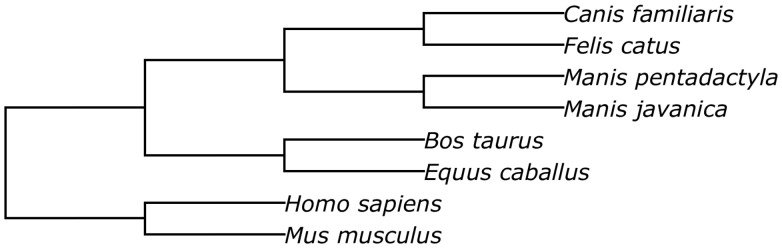
The phylogenetic tree of species involved into the structural alignment.

Prior to performing the structural alignment procedure, repetitive regions of the assemblies were masked with RepeatMasker (34). The phylogenetic tree used to perform the alignment is given in Figure 5.

### Pangolin genome conservation tracks

Basewise conservation scores were obtained for Malayan and Chinese pangolin genomes from the multispecies structural alignment using the HAL-phyloP tool from the HAL package (35). HAL-phyloP wraps the original phyloP algorithm (36) and is designed to efficiently process multispecies structural alignments by ProgressiveCactus.

Coding sequences (CDSs) of Malayan pangolin genes were used to train a neutral evolution model for phyloP. Next, phyloP was launched in the conservation and acceleration (‘CONACC’) mode to obtain the corresponding *P* values for each position in both pangolin genomes. The common logarithms of the basewise *P* values were reported; positions under acceleration were flagged by making their values negative, whereas positions under conservation had their values positive.

### Assembly hub for UCSC Genome Browser

The UCSC Genome Browser (37) is a well-known and widely used tool for bioinformatics analysis. Alongside with the PGD database, we provide an assembly hub (38) for the UCSC Genome Browser that contains datasets from PGD and provides means for viewing them in the genome browser. The hub also presents the structural alignment between genomes of both pangolin species and other mammals in the form of the snake tracks (39) and the genomic conservation tracks obtained from the alignment with the HAL-phyloP tool.

In addition to presenting the pangolin genome annotation tracks, the hub provides access to other features and tools available in the UCSC Genome Browser, including Table Browser (40) for manipulating the tracks and obtaining nucleotide sequences of the features.

### Data download

PGD provides a user-friendly interface for downloading pangolin genome and transcriptomic data and annotations. The raw sequencing reads from different library sizes are available for download. Users also can download the assembled reference genome sequences of the Malayan and Chinese pangolin for downstream analyses. Other available data or annotations are available for download including the MAKER-generated gene sequences (nucleotide CDS, exons and protein sequences), expressed genes from Malayan pangolin transcriptomes and the raw RNA-Seq data.

**Figure 6.**
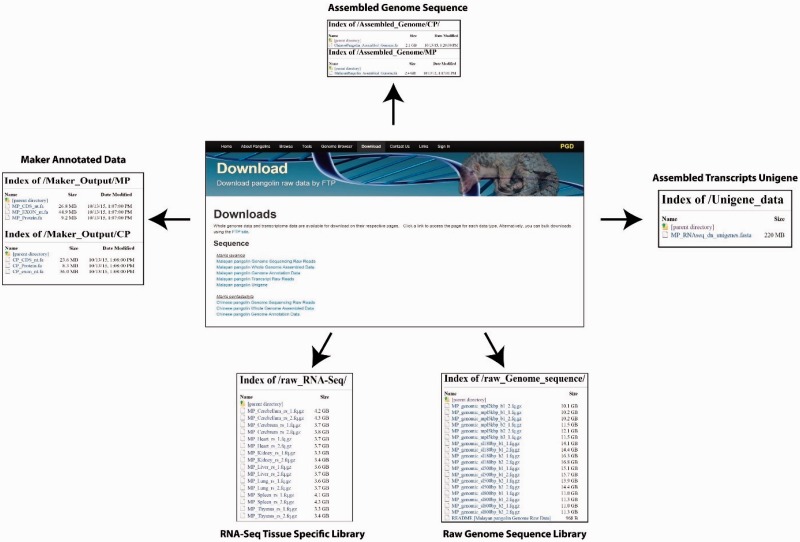
Web interfaces for data download in PGD.

There are two ways whereas users can download these data and annotations. First, users can download these data through the ‘Download’ page using the provided web interface. Users can select the data or annotations of interest to download. Second, users can download the raw data and annotations through the File Transfer Protocol interface provided in the download page (Figure 6).

## Conclusion

We anticipate that the PGD will become an invaluable hub, allowing researchers to access, browse, retrieve and analyze pangolin genomic and expression data and annotations. This hub would facilitate research in pangolin biology, particularly in the conservation of this critically endangered species and will also enhance our understanding of mammalian biology and evolution. We will continue updating PGD by incorporating more data, annotations and analysis tools particularly from our IPaRC consortium as it becomes available. We also welcome researchers to provide suggestions and/or share data for the improvement of the PGD hub.

## Availability

PGD is accessible at http://pangolin-genome.um.edu.my. The UCSC Genome Browser hub configuration file is available at http://public.dobzhanskycenter.ru/PangolinHub/hub.txt. Users can download all the raw sequences and datasets used in this paper from the PGD website.
